# Attention Bias in Individuals with Addictive Disorders: Systematic Review Protocol

**DOI:** 10.2196/resprot.9385

**Published:** 2018-02-08

**Authors:** Melvyn Zhang, JiangBo Ying, Guo Song, Roger CM Ho, Daniel SS Fung, Helen Smith

**Affiliations:** ^1^ Department of Family Medicine and Primary Care Lee Kong Chian School of Medicine Nanyang Technology University Singapore Singapore; ^2^ Institute of Mental Health Singapore Singapore; ^3^ Department of Psychological Medicine Yong Loo Lin School of Medicine National University of Singapore Singapore Singapore

**Keywords:** attention bias, substance addiction, systematic review

## Abstract

**Background:**

Globally, substance disorders, particularly that of opiate use, cannabis use, and stimulant use disorders, are highly prevalent. Psychological treatments are an integral aspect of intervention, but a proportion of individuals still relapse despite having received such an intervention. Recently, the dual-process theory proposed that the unconscious processes of attention biases are responsible for these relapses. Prior meta-analyses have reported the presence of attention bias in alcohol and tobacco use disorders. More recent research has examined attention bias and its effectiveness in opiate use, cannabis use, and stimulant use disorder. The evidence syntheses to date have not examined whether attention bias is present in these disorders and could be subjected to manipulation. This is important information and would support the introduction of psychological interventions for attention bias for such patients. Such psychological interventions would help individuals maintain their abstinence and minimize the risk of relapse.

**Objective:**

This paper aims to undertake a systematic review to synthesize the existing evidence for the presence of attention bias in all the disorders mentioned above, and to determine the clinical efficacy of attention bias modification.

**Methods:**

A systematic review will be conducted. A search will be conducted on the respective databases up till 2017. Selection of the studies will be determined by the Preferred Reporting Items for Systematic Review and Meta-Analysis guidelines. Quality assessment of the included studies will be assessed using the Cochrane Risk of Bias tool. A narrative synthesis will be conducted, with a meta-analysis considered only if there are sufficient studies for statistical analysis.

**Results:**

The results of the systematic review will be available 12 months after the publication of this protocol.

**Conclusions:**

This review is important as it will support the introduction of psychological interventions for attention bias for such patients. Such psychological interventions would help individuals maintain their abstinence and minimize the risk of relapse.

## Introduction

### Background

The recent statistics from the United Nations Office on Drugs and Crime reported that a quarter of a billion individuals experimented with drugs in 2015, and 29.5 million individuals are currently diagnosed with a substance disorder [1]. The most commonly abused drugs include cannabis, opioids and amphetamines [1] with a prevalence of 3.8%, 0.7% and 0.77%, respectively [1]. Drug usage and dependence is also associated with comorbidities, such as that of retroviral diseases and hepatitis C. Among the 12 million individuals who have used drugs intravenously, 1.6 million individuals have acquired a retroviral disease and another 6.1 million individuals have acquired hepatitis C [1]. In response to this huge problem, the World Health Organization and the United Nations Office on Drugs and Crime have been working jointly in the formulation of various policies, and to improve the existing levels of care in low- and middle-income countries [2].

The treatment options for drug abuse are both pharmacological and nonpharmacological. Psychosocial treatments complement and augment the effectiveness of pharmacological treatments, and prior reviews have reported the effectiveness of a combination treatment for opioid use disorders [3]. A variety of psychological approaches are used, such as contingency management and cognitive behavioural therapy (CBT). While therapies such as CBT have been demonstrated to be efficacious in the treatment of cannabis use disorder [4], other studies have reported that despite its effectiveness, at least 40%-50% of individuals relapse within a year of successful treatment, and 70% of individuals relapse within three years [5]. Pharmacological options for the treatment of addictive disorders are varied; for cannabis and amphetamine disorders, these are limited, whereas for opioid use disorders, opiate substitution therapies (eg, methadone and buprenorphine) can be used [6]. Current evidence supports the use of symptomatic medications in the management of individuals who are acutely intoxicated, such as benzodiazepines for its sedative properties, and antipsychotics for individuals experiencing psychotic symptoms [7]. Recent research has addressed the use of gabapentin in the treatment of cannabis withdrawal, but further trials are necessary to determine its efficacy [7].

The observed high relapse rates into substance usage suggest that current psychosocial interventions do not completely address the issues leading to either lapse or relapse. The dual process model of addiction [8,9] has been posited to explain the lapse and relapses; this model proposes the involvement of two disparate processes, the reflective and the automatic process [8]. The reflective process involves conscious decision-making, and this has been the target of CBT treatment. Automatic processes refer to that of attentional bias [10], which are unconscious processes that lead individuals to focus on substance-related cues in their naturalistic environment and, having done so, experience a corresponding difficulty in disengaging from cues [10]. Such an automatic process increases the salience of substance-related cues and could potentially draw abusers towards substances. Attention bias could be assessed using indirect measures, like that of Stroop test or a Visual Probe task, or the use of direct measures through eye movement tracking [10]. Indirect measures have the potential to be incorporated into an attention bias modification task, to help individuals cope with automatic processes that increase their intrinsic risk for relapse [11].

To date, there have been several studies that have evaluated the efficacy of attention bias modification paradigms for addictive disorders. Cristea et al [12] undertook a meta-analysis of attention bias modification for alcohol and tobacco disorders and found no significant effect of cognitive bias modification on craving and addiction outcomes, even though there was a moderate effect on cognitive bias. Their findings are important as they demonstrate the presence of cognitive bias. In addition, attention bias modification was effective against such biases. Unfortunately, Cristea et al’s [12] meta-analysis was unable to demonstrate significant changes in secondary outcomes, but this could be because more follow-up time is required before a change in symptomatology is detectable. This review was further limited by the high risk of bias amongst the studies included and its focus being limited to alcohol or tobacco. Christiansen et al’s [13] review had a wider target, including individuals who were using alcohol, tobacco, cocaine and cannabis. They attempted to appraise the existing evidence to determine whether attention bias assessed in treatment settings are predictive of relapse and whether modifying attention bias can help improve outcomes. Their review found mixed evidence. This study, however, had a number of limitations: the intrinsic methodological weaknesses of the included studies, the lack of clarity about inclusion and exclusion criteria, the time frame during which the search was conducted, and using search results from only PubMed and Scopus.

To date, there has been research that has examined attention bias and determined the effectiveness of attention bias modification in other addictive disorders, like opiate use disorder [14], cannabis use disorder [15] as well as amphetamine or stimulant use disorder [16]. However, there has been no evidence synthesis of these studies. There is a need to synthesize the information about attention bias and bias modification among opiate use, cannabis use and stimulant use disorder. This gap needs addressing urgently as these substances are being increasingly utilized globally. It will also be pertinent to determine the efficacy of attention bias modification interventions among these disorders.

### Review Aims

The primary aim of this systematic review is to synthesize the current evidence with regards to attention bias among opioid use, cannabis use and stimulant use disorders. As a secondary aim, it will determine the efficacy of attentional bias modification interventions, including attentional bias reduction, cravings score and addiction outcomes. These will be correlated with the methodology that is being utilized in attention bias assessment and modification.

We will undertake a systematic review to synthesize the existing evidence for the presence of attention bias in the disorders mentioned above, and to determine the clinical efficacy of attention bias modification. The studies will be selected by independent assessors and screened against our inclusion and exclusion criteria. A risk of bias assessment will be conducted to assess the quality of the included studies.

If there are sufficient studies, a meta-analytical review will be conducted to determine the effect size of attention bias modification intervention for opioid use, cannabis use and stimulant use disorders.

## Methods

### Search Strategy

To identify the relevant articles, search terminologies as outlined in Textbox 1 will be used. The search terms will be combined using the Boolean operator “OR” and the search terms between two disparate concepts will be combined using the Boolean operator “AND”.

A comprehensive search will be conducted on the following databases: PubMed, MEDLINE, Embase, PsycINFO, Science Direct, Cochrane CENTRAL and Scopus. If full-text access is not available, the original authors will be contacted for their articles. Proceedings from scientific meetings and conference abstracts will also be included.

### Inclusion and Exclusion Criteria

Only articles written in English will be included. The inclusion criteria are as follows: (a) attention bias assessed using a validated measure (such as that of Stroop test or Visual Probe/Dot-Probe Task); (b) participants in the studies must have a primary diagnosis of opiate use, cannabis use or stimulant use disorder; and (c) the study design must be a randomized trial. Studies will be excluded if (a) they have not included a validated measure for the assessment of attention bias (such as Stroop Test or Visual Probe/Dot-Probe Task); (b) participants in the studies have been diagnosed with another mental health disorders as their primary disorder (eg, depression as the primary disorder and substance use disorder as the secondary diagnosis); (c) studies involved a pharmacological intervention in which medications were utilized to determine their effects on attention bias; and (d) the randomized trial involved a cross-over design (given the high risk of bias associated with a cross-over design).

### Condition or Domain Being Studied

This systematic review focuses on substance use disorders, and in particular, opioid use, cannabis use and stimulant use disorder.

### Participants

Participants must be diagnosed with a substance use disorder, that of opioid use, cannabis use or stimulant use disorder, as the main or primary disorder. Participants may include individuals from the general population or a treatment-seeking cohort, and can be adolescent or adult.

### Intervention / Exposure

The intervention administered to participants is either that of a Stroop or Visual Probe/Dot-Probe attention modification task.

### Comparison with Placebo Group

Individuals may be compared with individuals who have received a placebo training or sham training interventions or individuals who have received only normal routine care.

### Outcome

For the primary aim, the outcome will be the presence of attention bias as measured using a validated assessment tool. Attention bias is deemed to be present if participants are noted to have a longer reaction time spent on drug-related stimuli as compared to neutral stimuli.

For the secondary aim, the outcomes will be: a) reduction in the mean reaction time following the attention bias modification intervention; b) score reductions on validated craving measures (either a single-dimensional score, or a visual analogue scale or a multidimensional score such as that of the obsessive-compulsive craving score); and c) addiction outcomes, such as the mean time to relapse or the time maintained in abstinence.

### Data Extraction, Sorting and Selection

The search strategy will identify articles that may have potential relevance. Selection of relevant publications will be conducted independently by two authors (MWBZ and JY). Articles will be first screened based on their title and abstract. Those shortlisted will be evaluated against the inclusion and exclusion criteria. Any disagreement between the two reviewers will be resolved through a discussion with the third author. An electronic form will be utilized to record the reasons for the inclusion and exclusion of each article. The current systematic review protocol will adhere to the reporting guidelines of the Preferred Reporting Items for Systematic Reviews and Meta-Analysis Protocols [17].

The following data and information will be extracted from each article, recorded on a standardized electronic data collation form and cross-checked by the second author:

1. Publication details: author(s) and study year.
2. Study design and methodology: study design, sample size (intervention and control group), types of sample (treatment seeking or individuals in general population), country in which study was conducted, demographics of sample (mean age, age range, proportion of males and females), diagnosis of participants (opioid use, cannabis use or stimulant use disorder), methodology in which diagnosis is made.
3. Attention bias assessment and modification methodology: types of attention bias tools utilized (Stroop test or Visual Probe task).
4. Outcomes of interest: craving scores (as assessed using a validated questionnaire or toolkit), addiction outcomes (time to next relapse, amount of substances used), effect size (Cohen’s *d* or Hedges’ *g*) for attention bias modification procedure.

**Search terminologies.**

(“attention bias” OR “approach bias” OR “avoidance bias” OR “cognitive bias”) AND (“addiction” OR “substance” OR “drug” OR “abuse” OR “Dependence” OR “Alcohol” OR “Drinking” OR “Opiates” OR “Heroin” OR “Cannabis” OR “Marijuana” OR “Stimulants” OR “Amphetamines” OR “Cocaine”)

### Quality Assessment

For the risk of bias assessment, the Cochrane Risk of Bias tool [18] will be used.

### Strategy for Data Integration and Synthesis

For the systematic review, we will synthesize and report whether attention bias was present and how its presence was determined. We will also synthesize the findings of the studies narratively and report whether attention bias modification was effective.

If there are sufficient studies for each of the conditions, a meta-analysis will be conducted to synthesize statistically the pooled effect size for attention bias modification for opiate use disorder, cannabis use and stimulant use disorders. For the meta-analytic study, the statistical analysis will be performed using Comprehensive Meta-Analysis Version 2.0 based on the random-effects model. The random effects model assumes that there are varying effect sizes between the studies, due to the underlying differences in study designs and intrinsic heterogeneity of the sampled populations. The statistical analysis will compute the pooled effect size to determine the clinical efficacy of attention bias modification and to identify potential moderators (both categorical and continuous variables) that could account for the heterogeneity in the effect size computed. Between-study heterogeneity will be assessed with the I^2^ statistic, which describes the percentage of variability among effect estimates beyond that expected by chance. As a reference, I^2^ values of 25% are considered low, 50% moderate and 75% high in heterogeneity. Meta-regression analysis will be conducted to identify demographic variables that could contribute to the heterogeneity and the effect size computed. The regression coefficients and the associated *Z* and *P* values will be reported. Subgroup analysis will be undertaken to investigate the effects of categorical variables on the effect size obtained. For the meta-analysis, Egger’s regression test will be conducted to determine if publication bias is present. If there is significant publication bias, the classic fail-safe test will be performed to determine the number of missing studies that will be required for the *P* value of the publication bias to be higher than .05.

## Results

We expect that the review will be completed 12 months from the publication of this protocol. We will report the results based on the identified outcomes as specified above.

## Discussion

We are aware of the prior reviews investigating the efficacy of attention bias modification among substance use disorders, but these have been limited by the inclusion of select studies involving cohorts who have either alcohol use or tobacco use disorders. As there is a proliferation of research examining attention bias and bias modification among other highly prevalent substance use disorders, particularly that of opiate use, cannabis use and stimulant use disorders, there is a need to synthesize the evidence for attention bias and attention bias modification for these disorders. It is important to establish that attention bias is present in these disorders, and that it could be amenable to modification using conventional paradigms such as the Stroop testing or Visual Probe task.

The findings of this proposed review will have important clinical implications. Should attention biases be found among individuals with opioid, cannabis and stimulant use disorder, clinicians will need to review their treatment strategies. Rather relying on a single modality of therapy to modify conscious control, they will need to augment the conventional psychological interventions with one that targets attention bias. The current review will also determine which indirect method of bias modification is more efficacious for bias modification in substance using cohorts.

## Abbreviations

CBT: cognitive behavioural therapy

## References

[1] (2017). United Nations Office of Drugs Crime.

[2] (2017). World Health Organization.

[3] Dugosh K, Abraham A, Seymour B, McLoyd K, Chalk M, Festinger D (2016). A Systematic Review on the Use of Psychosocial Interventions in Conjunction With Medications for the Treatment of Opioid Addiction. J Addict Med.

[4] Hofmann S, Asnaani A, Vonk I, Sawyer A, Fang A (2012). The efficacy of cognitive behavioral therapy: a review of meta-analyses. Cognit Ther Res.

[5] Cutler Robert B, Fishbain David A (2005). Are alcoholism treatments effective? The Project MATCH data. BMC Public Health.

[6] (2009). World Health Organization.

[7] Gorelick D (2016). Pharmacological Treatment of Cannabis-Related Disorders: A Narrative Review. Curr Pharm Des.

[8] Boffo M, Pronk T, Wiers R, Mannarini S (2015). Combining cognitive bias modification training with motivational support in alcohol dependent outpatients: study protocol for a randomised controlled trial. Trials.

[9] Heitmann J, van HM, Vermeulen K, Ostafin B, MacLeod C, Wiers R, DeFuentes-Merillas Laura, Fledderus Martine, Markus Wiebren, de Jong Peter J (2017). Internet-based attentional bias modification training as add-on to regular treatment in alcohol and cannabis dependent outpatients: a study protocol of a randomized control trial. BMC Psychiatry.

[10] Field M, Cox W (2008). Attentional bias in addictive behaviors: a review of its development, causes, and consequences. Drug Alcohol Depend.

[11] Boendermaker W, Sanchez MS, Boffo M, Wiers R (2016). Attentional Bias Modification With Serious Game Elements: Evaluating the Shots Game. JMIR Serious Games.

[12] Cristea I, Kok R, Cuijpers Pim (2016). The Effectiveness of Cognitive Bias Modification Interventions for Substance Addictions: A Meta-Analysis. PLoS One.

[13] Christiansen P, Schoenmakers T, Field Matt (2015). Less than meets the eye: reappraising the clinical relevance of attentional bias in addiction. Addict Behav.

[14] Bearre L, Sturt P, Bruce G, Jones BT (2007). Heroin-related attentional bias and monthly frequency of heroin use are positively associated in attenders of a harm reduction service. Addict Behav.

[15] Cousijn J, Watson P, Koenders L, Vingerhoets WAM, Goudriaan AE, Wiers RW (2013). Cannabis dependence, cognitive control and attentional bias for cannabis words. Addict Behav.

[16] Morgan CJA, Rees H, Curran HV (2008). Attentional bias to incentive stimuli in frequent ketamine users. Psychol. Med.

[17] Moher D, Shamseer L, Clarke M, Ghersi D, Liberati A, Petticrew M, Shekelle P, Stewart LA, PRISMA- P (2015). Preferred reporting items for systematic review and meta-analysis protocols (PRISMA-P) 2015 statement. Syst Rev.

[18] Higgins J, Altman DG, Gotzsche PC, Juni P, Moher D, Oxman AD, Savovic J, Schulz KF, Weeks L, Sterne JAC (2011). The Cochrane Collaboration's tool for assessing risk of bias in randomised trials. BMJ.

